# Surgical and demographic predictors of free flap salvage after takeback: A systematic review

**DOI:** 10.1002/micr.30921

**Published:** 2022-05-25

**Authors:** Scott K. Odorico, Katie Reuter Muñoz, Peter J. Nicksic, Kirsten A. Gunderson, Kasey Wood, Zeeda H. Nkana, Evalina Bond, Samuel O. Poore

**Affiliations:** ^1^ Division of Plastic Surgery, Department of Surgery University of Wisconsin‐Madison, School of Medicine and Public Health Madison Wisconsin USA

## Abstract

**Background:**

Microsurgical free tissue transfer (FTT) is a widely employed surgical modality utilized for reconstruction of a broad range of defects, including head and neck, extremity, and breast. Flap survival is reported to be 90%–95%. When FTT fails, salvage procedures aim at establishing reperfusion while limiting ischemia time—with salvage rates between 22% and 67%. There are limited data‐driven predictors of successful salvage present in the literature. This systematic review aims to identify predictors of flap salvage.

**Methods:**

A systematic literature review was conducted per PRISMA guidelines. Articles included in the final analysis were limited to those investigating FTT salvage procedures and included factors impacting outcomes. Cohort and case series (>5 flaps) studies up until March 2021 were included. Chi‐square tests and linear regression modeling was completed for analysis.

**Results:**

The patient‐specific factors significantly associated with salvage included the absence of hypercoagulability (*p* < .00001) and no previous salvage attempts (*p* < .00001). Case‐specific factors significantly associated with salvage included trunk/breast flaps (*p* < .00001), fasciocutaneous/osteocutaneous flaps (*p* = .006), venous compromise (*p* < .00001), and shorter time from index procedure to salvage attempt (*R* = .746). Radiation in the head and neck population was significantly associated with flap salvage failure.

**Conclusions:**

Given the complexity and challenges surrounding free flap salvage procedures, the goal of this manuscript was to present data helping guide surgical decision‐making. Based on our findings, patients without documented hypercoagulability, no previous salvage attempts, fasciocutaneous/osteocutaneous flaps, trunk/breast flaps, and a shorter time interval post‐index operation are the best candidates for a salvage attempt.

## 1 INTRODUCTION

Microsurgical free tissue transfer (FTT) is a widely employed operation utilized for reconstruction of various defects. FTT can be used for wound coverage in various anatomic locations, including the head/neck, lower and upper extremity, and breast (Cho et al., 2018; Eckart & Fokas, 2003; Gill et al., 2004). Survival of FTT is reported to be between 90% and 97% (Cho et al., 2018; Eckart & Fokas, 2003; Gill et al., 2004; Yang et al., 2014; Zhao et al., 2020), making it a reliable tool for the reconstructive surgeon. However, complications do arise, which can present as venous or arterial thrombosis, hematoma, or infection. Most commonly, thrombosis or hematoma can result in acute flap compromise, requiring salvage operation—a procedure involving exploration of the anastomosis, thrombectomy, thrombolysis, re‐do of the anastomosis, or combinations (Ho et al., 2012; Pu et al., 2004). Based on the literature, FTT salvage procedure success is reported to range from 22% to 67% (Mirzabeigi et al., 2012; Saint‐Cyr et al., 2007; Tall et al., 2015; Wang et al., 2019), which necessitates the exploration of nuances which predict FTT salvage.

Efforts to maximize success of salvage procedures manifest as clinical monitoring following primary FTT. Most commonly, ancillary staff perform clinical assessments of the flap—noting characteristics such as color, turgor, and capillary refill—as well as Doppler ultrasonography to assess adequate perfusion and drainage of the flap (Abdel‐Galil & Mitchell, 2009; Disa et al., 1999). This system of flap monitoring is rooted in the knowledge that salvage success rates drop precipitously as time between FTT and return to the operating room (RTOR) increases (Smit et al., 2007). While clinical monitoring is standard of care for most institutions, it remains a costly endeavor and does not eliminate salvage failure (Jablonka et al., 2019). Shen et al. reviewed 11 studies and showed that salvage rates decrease by postoperative day and anatomic location, with breast flaps having the highest salvage rates (Shen et al., 2021).

To our knowledge, no systematic reviews or meta‐analyses have examined patient‐ and operation‐specific factors which impact FTT salvage outcomes other than time and anatomic location. Therefore, the goals of this study were to conduct a systematic review of the literature to (1) identify any factors that impact FTT salvage outcomes and (2) aid the reconstructive surgeon in counseling patients on chances of a successful salvage.

## 2 MATERIALS AND METHODS

### 2.1 Search strategy

A systematic review of publications evaluating FTT salvage outcomes and factors associated with outcomes was conducted using the preferred reporting items for systematic reviews and meta‐analyses (PRISMA) guidelines (Moher et al., 2009). PubMed, Scopus, and Web of Science were queried without date of publication restrictions. Search terms included: “free flap OR free tissue OR free tissue transfer OR flap reconstruction OR microvascular flap OR DIEP flap OR head and neck flap OR lower extremity free flap” AND “salvage OR salvage outcome OR take back OR take‐back OR compromise OR failure” AND “predictor OR risk factor OR factor OR influence OR comorbidity.”

### 2.2 Inclusion and exclusion criteria

Inclusion criteria were cohort studies and case series (greater than five patients) that included data involving FTT salvage procedures of any anatomic site and case‐ or patient‐specific factors impacting outcomes in humans. Exclusion criteria were studies reporting salvage rate without factors influencing outcomes, case reports, case series with less than five patients, studies in press or unpublished meeting proceedings, nonhuman studies, and non‐English studies.

### 2.3 Article selection

After obtaining records following our search query, all titles were screened independently by two authors (SKO, KRM) for relevance based on a priori criteria. Screened articles were then selected for full‐text review, which were completed by two authors (SKO, KRM) with additional screening from supporting authors. At every stage, articles required two authors to agree regarding inclusion. If a disagreement occurred, the senior author was consulted.

### 2.4 Data collection and analysis

Data extraction utilized a standardized form for accuracy. Data collection included authors, journal, publication year, total salvage attempts, total salvage success, and number of salvage attempts and success for each anatomic group (head/neck, extremity, and trunk/breast). Additionally, for categorical data, we collected the total number of successful or failed salvage attempts for patients with and without the following demographic information: gender (male/female), smoking status, hypertension (HTN), diabetes mellitus (DM), high cholesterol, peripheral vascular disease and coronary artery disease (PVD/CAD), hypercoagulability (defined as thrombophilia or previous thromboembolic event), previous radiation, previous chemotherapy, and previous takebacks. We collected the number of successful and failed salvages based on venous or arterial compromise, venous supercharging (>1 venous anastomosis), and tissue type—osteocutaneous, fasciocutaneous, or myocutaneous. For continuous data, we collected average study values for both successful and unsuccessful salvages for the following variables: age, body mass index (BMI), time from index operation to compromise detection (i.e., clinical identification, typical by Doppler probe), and total time from index operation to salvage.

Successful salvage was defined as both complete and partial salvages. As Mirzabeigi et al. described, partial flap loss and fat necrosis etiology are multifactorial, including factors like perforator selection, and not in the scope of this review (Mirzabeigi et al., 2012). Statistical analysis was completed in Microsoft Excel (Microsoft, Redmond, WA) using chi‐squared tests for categorical variables and correlation coefficients for continuous variables reported in Pearson's correlation coefficient and linear regression analysis *p‐*values. Statistical significance was set at *p‐*values <.05.

## 3 RESULTS

**FIGURE 1:**
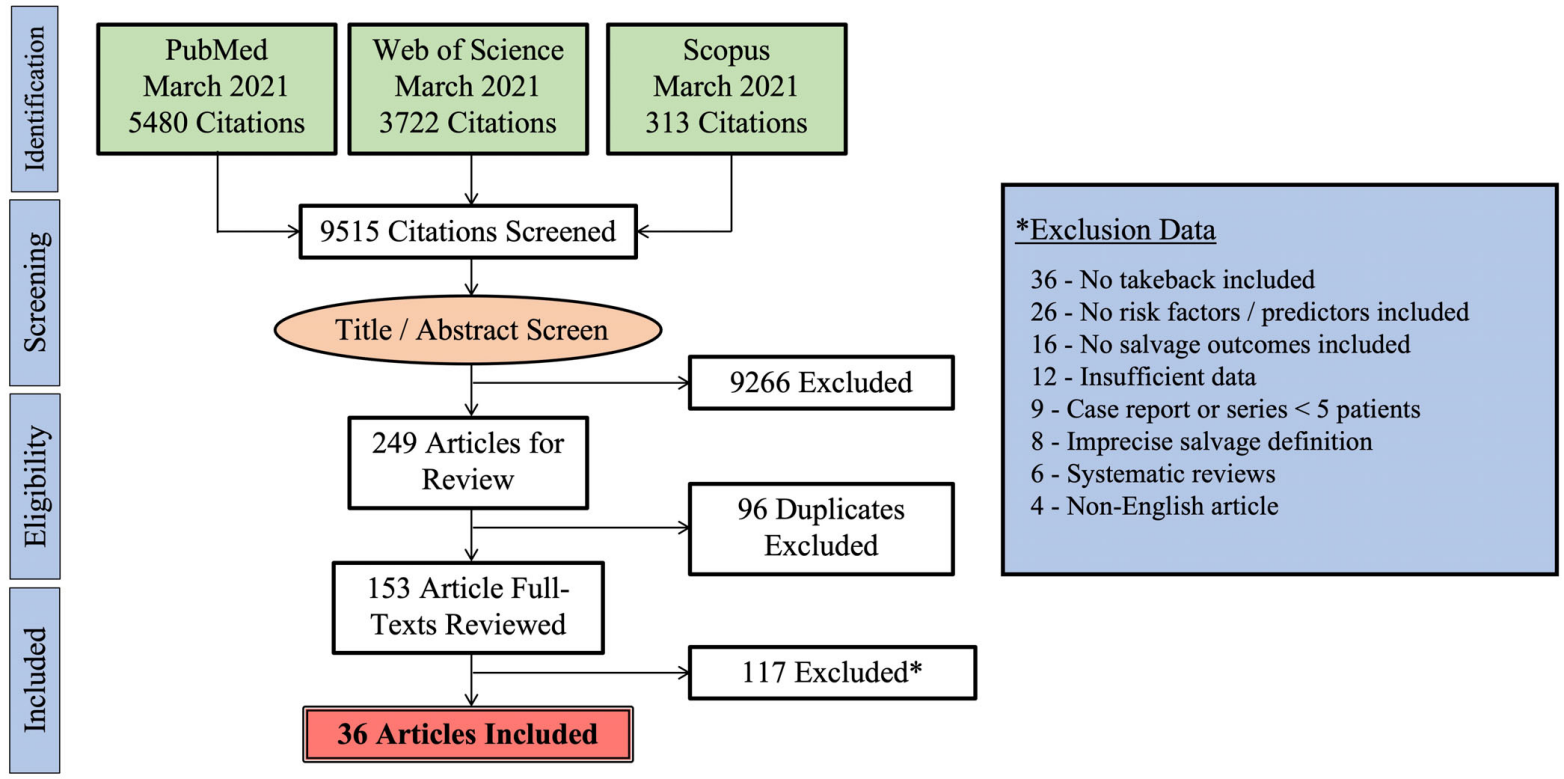
PRISMA guidelines for systematic review flow chart summarizing our initial article search and selection process.

After our initial query, 9515 articles underwent title and abstract review (Figure 1). Deduplication occurred for 249 studies, and 153 records underwent full‐text review. After applying inclusion and exclusion criteria, 36 articles were included for analysis (Table 1). The date range for the studies included was June 2001 to March 2021. There were 1840 successful FTT salvages in 2735 total attempts, resulting in an average salvage rate of 67.28% (Table 2). While 11 studies (30.56%) did not mention any monitoring protocol, Doppler ultrasonography was used in 18 studies (50.00%) with 7 studies (19.44%) mentioning clinical monitoring without specific mention of ultrasonography. Overall, average salvage rates varied by anatomic location: head/neck (57.81%), trunk/breast (71.25%), extremity (50.76%). Salvage rates also varied by tissue type: fasciocutaneous (67.24%), osteocutaneous (58.97%), and myocutaneous (52.41%).

**TABLE 1:** Free flap salvage systematic review articles.

Author	Year	Journal	*N*	Salvage rate	Author	Year	Journal	*N*	Salvage rate
Brown et al.	2013	*Brit Journ of Oral Maxillofac Surg*	40	72.50%	Liu et al.	2018	*Laryngoscope*	7	14.29%
Bui et al. (Bui et al., 2007)	2007	*PRS*	38	63.16%	Mirzabeigi et al. (Mirzabeigi et al., 2012)	2012	*PRS*	47	48.94%
Carney et al. (Carney et al., 2018)	2018	*PRS*	70	65.71%	Nakamizo et al.	2004	*Auris Nasus Larynx*	7	28.57%
Chang et al. (Chang, Zhang, et al., 2016)	2016	*Head & Neck*	151	60.26%	Selber et al. (Selber et al., 2012)	2012	*PRS*	157	57.96%
Chang et al. (Chang, Chang, et al., 2016)	2016	*Ann of Plast Surg*	166	73.49%	Smit et al. (Smit et al., 2007)	2007	*Microsurgery*	69	62.32%
Chen et al. (Chen et al., 2007)	2007	*PRS*	113	84.07%	Stranix et al.	2018	*PRS*	71	47.89%
Chen et al.	2012	*Microsurgery*	9	77.78%	Sweeny et al. (Sweeny et al., 2020)	2020	*Head & Neck*	162	45.68%
Chiu et al. (Chiu et al., 2017)	2017	*Ann of Plast Surg*	150	72.67%	Tall et al.8	2015	*Ann of Plast Surg*	27	22.22%
Cho et al. (Cho et al., 2018)	2018	*PRS*	70	51.43%	Wang et al. (Wang et al., 2012)	2012	*PRS*	12	33.33%
Choi et al.	2019	*Journ of Reconstr Microsurg*	36	77.78%	Wang et al. (Wang et al., 2019)	2019	*Microsurgery*	21	61.90%
Dassonville et al.	2008	*Euro Arch Oto*	32	65.63%	Wettstein et al.	2008	*Journ Plast Reconstr Aesth Surg*	13	38.46%
Ho et al. (Ho et al., 2012)	2012	*Brit Journ of Oral Maxillofac Surg*	72	59.72%	Winterton et al. (Winterton et al., 2010)	2010	*Journ Plast Reconstr Aesth Surg*	327	88.99%
Hyodo et al. (Hyodo et al., 2007)	2007	*Laryngoscope*	21	33.33%	Yang et al. (Yang et al., 2014)	2014	*Int Journ Oral Maxillofac Surg*	47	55.32%
Joseph et al.	2021	*Euro Journ of Plast Surg*	43	44.19%	Yang et al.	2016	*Int Journ Oral Maxillofac Surg*	71	66.20%
Kamali et al.	2021	*Journ Plast Reconstr Aesth Surg*	62	70.97%	Yii et al. (Yii et al., 2001)	2001	*Ann of Plast Surg*	41	68.29%
Khansa et al.	2013	*Microsurgery*	113	84.96%	Yim et al.	2015	*Arch Plast Surg*	17	82.35%
Las et al.	2016	*Journ Plast Reconstr Aesth Surg*	100	77.00%	Yu et al.	2008	*Head & Neck*	49	44.90%
Lee et al. (Lee & Mun, 2013)	2013	*Journ Plast Reconstr Aesth Surg*	31	58.06%	Zhao et al. (Zhao et al., 2020)	2020	*Journ of Reconstr Microsurg*	273	74.73%

Abbreviation: PRS: plastic and reconstructive surgery.

**TABLE 2:** Free flap salvage rates summary.

		Successful/attempts (%)
Overall average salvage rate		1840/2735 (67.28)
**Overall anatomic salvage rates**		
Head and neck		596/1031 (57.81)
Trunk/breast		389/546 (71.25)
Extremity		100/197 (50.76)
**Overall flap type salvage rates**		
Fasciocutaneous		78/116 (67.24)
Osteocutaneous		23/39 (58.97)
Myocutaneous		98/187 (52.41)

### 3.1 Patient‐specific factors

**FIGURE 2:**
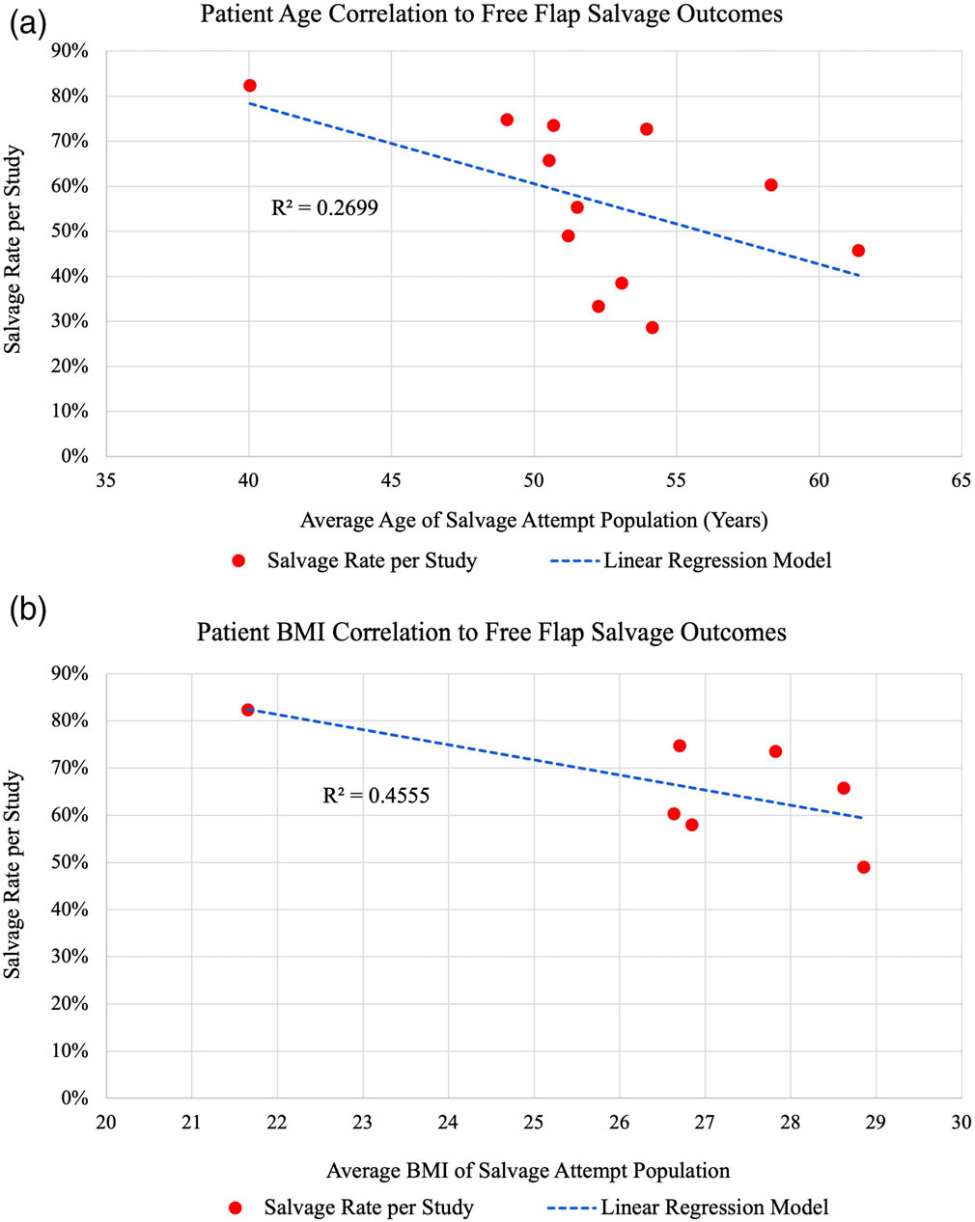
Linear regression model on scatter plots for average (a) patient age and (b) patient BMI per study correlated with study salvage rate. Age is negatively correlated with salvage rate (*R* = −.5195), as is BMI (*R* = −.6749), but neither are statistically significant (*p* = .083 and .096, respectively). Alpha was set at .05.

The presence of hypercoagulability in patients was associated with higher failure rates in free flap salvage procedures (Table 3). Radiation history was not associated with free flap salvage failure but was significantly associated with flap salvage failure in the head and neck portion of flaps (*X* ^2^ = 4.776, *p* = .028, *N* = 646; data not shown). Previous takebacks were significantly associated with free flap salvage failure. Patient age (*R* = −.5195) and BMI (*R* = −.6749) were both negatively correlated with salvage rate, but neither were statistically significant (*p* = .083 and *p* = .096, respectively) (Figure 2a,b). Other patient‐specific factors which were assessed but not significantly associated with salvage outcomes included HTN, gender, DM, smoking, PVD/CAD, prior chemotherapy, and high cholesterol (Tables 3 and 4).

**TABLE 3:** Factors associated with salvage outcomes, part 1.

Hypercoagulability			Previous takeback		
	Success	Failure		Success	Failure
Hypercoagulability	11	31	Previous takeback	25	54
No hypercoagulability	153	85	No previous takeback	157	72
Chi‐squared stat		21.3513	Chi‐squared stat		33.1085
*p*‐value		<.00001	*p*‐value		<.00001
Significance level		.05	Significance level		.05
*N*		280	*N*		308

Radiation			Hypertension		
	Success	Failure		Success	Failure
Radiation	187	140	Hypertension	184	72
No radiation	381	221	No hypertension	322	162
Chi‐squared stat		3.3214	Chi‐squared stat		2.2132
*p*‐value		.068384	*p*‐value		.136835
Significance level		.05	Significance level		.05
*N*		929	*N*		740

**TABLE 4:** Factors associated with salvage outcomes, part 2.

Gender				Diabetes mellitus				Smoking		
	Success	Failure			Success	Failure			Success	Failure
Male	239	157		DM	48	30		Smoking	300	167
Female	109	90		No DM	423	251		No smoking	402	199
Chi‐squared stat		1.6983		Chi‐squared stat		0.0445		Chi‐squared stat	0.8185	
*p*‐value		.192515		*p*‐value		.832842		*p*‐value		.365626
Significance level		.05		Significance level		.05		Significance level		.05
*N*		595		*N*		752		*N*		1068

PVD/CAD				Prior chemotherapy				High cholesterol		
	Success	Failure			Success	Failure			Success	Failure
PVD/CAD	55	24		Chemo	103	69		High chol	52	18
No PVD/CAD	319	201		No chemo	175	112		No chol	221	99
Chi‐squared stat		2.0019		Chi‐squared stat		0.0537		Chi‐squared stat		0.7462
*p*‐value		.157101		*p*‐value		.81677		*p*‐value		.38769
Significance level		.05		Significance level		.05		Significance level		.05
*N*		599		*N*		459		*N*		390

Abbreviations: Chemo: chemotherapy; Chol: cholesterol; PVD/CAD: peripheral vascular disease/coronary artery disease.

### 3.2 Surgery‐specific factors

**FIGURE 3:**
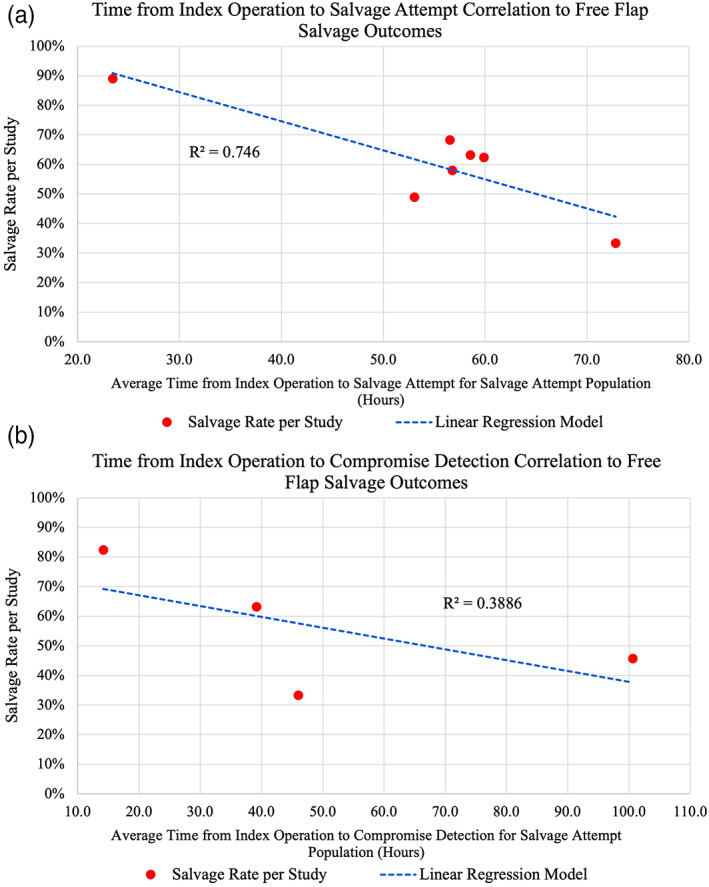
Linear regression model on scatter plots for average time from index operation to (a) salvage attempt and (b) compromise detection. Both are negatively correlated with salvage rates, but only time from index operation to salvage attempt was statistically significant (*p* = .012). Alpha was set at .05.

For anatomic location of flaps, breast/trunk free flaps were significantly associated with successful salvage outcomes compared with both head/neck flaps, as well as extremity flaps (Table 5). When comparing head/neck free flaps to extremity free flaps, there was no significant difference in associated salvage rates. The time from index operation to salvage attempt was negatively correlated with salvage rate (*R* = −.8637, *p* = .012) (Figure 3a). The average times from index operation to salvage attempt for the failure and success groups were 78.19 h (56.1–100.8) and 37.75 h (19.4–46.5), respectively. A similar relationship was found when correlating time from index operation to compromise detection (*R* = −.6234, 59.08 h vs. 30.04 h for failure and success groups, respectively), but this was not significant (*p* = .38) (Figure 3b).

**TABLE 5:** Factors associated with salvage outcomes, part 3.

Breast versus H&N			Breast versus extremity		
	Success	Failure		Success	Failure
Breast	389	157	Breast	389	157
H&N	596	435	Extremity	100	97
Chi‐squared stat		27.4891	Chi‐squared stat		26.9982
*p*‐value		<.00001	*p*‐value		<.00001
Significance level		.05	Significance level		.05
*N*		1577	*N*		743

Venous versus arterial			Venous versus combined		
	Success	Failure		Success	Failure
Venous	518	212	Venous	518	212
Arterial	259	198	Combined	36	100
Chi‐squared stat		25.3654	Chi‐squared stat		98.4481
*p*‐value		<.00001	*p*‐value		<.00001
Significance level		.05	Significance level		.05
*N*		1187	*N*		866

H&N versus extremity			Fasciocutaneous versus myocutaneous		
	Success	Failure		Success	Failure
H&N	596	435	Fasciocutaneous	192	75
Extremity	100	97	Myocutaneous	171	115
Chi‐squared stat		3.3447	Chi‐squared stat		8.9937
*p*‐value		.067423	*p*‐value		.002709
Significance level		.05	Significance level		.05
*N*		1228	*N*		553

Arterial versus combined			Myocutaneous versus osteocutaneous		
	Success	Failure		Success	Failure
Arterial	259	198	Myocutaneous	171	115
Combined	36	100	Osteocutaneous	33	20
Chi‐squared stat		38.2457	Chi‐squared stat		0.1142
*p*‐value		<.00001	*p*‐value		.7354
Significance level		.05	Significance level		.05
*N*		593	*N*		339

Fasciocutaneous versus osteocutaneous			Myocutaneous versus osteocutaneous/fasciocutaneous		
	Success	Failure		Success	Failure
Fasciocutaneous	192	75	Myocutaneous	171	115
Osteocutaneous	33	20	Osteo/fascio	225	95
Chi‐squared stat		1.9712	Chi‐squared stat		7.3841
*p*‐value		.160325	*p*‐value		.00658
Significance level		.05	Significance level		.05
*N*		320	*N*		606

Abbreviation: H&N: head and neck.

Venous compromise was associated with a higher percentage of free flap salvage when compared to arterial compromise (Table 5). When compared to a combined venous and arterial compromise etiology, both venous alone and arterial alone were associated with higher flap salvage rates. When analyzing flap tissue type, fasciocutaneous free flaps were associated with a significantly higher salvage rate when compared with myocutaneous FTT (Table 5). This relationship resembles when we combine osteocutaneous and fasciocutaneous flaps and compare this to muscle‐containing flaps, with muscle‐containing flaps being associated with lower salvage rates (Table 5). Lastly, venous supercharging was not associated with higher flap salvage rates (*p* = .122), regardless of the anatomic location (H/N: *p* = .105; trunk/breast: *p* = .262; data not shown).

## 4 DISCUSSION

The present systematic review is the first to summarize and analyze the current literature's data regarding both patient‐ and case‐specific factors impacting free flap salvage following FTT compromise. This investigation yielded 36 peer‐reviewed articles analyzing free flap salvage outcomes. Of the novel, patient‐specific findings assessed in our review, hypercoagulability and previous takeback attempts were most significantly associated with lower salvage rates. Of the case‐specific findings, increased time from index operation to salvage attempt and from index operation to detection were highly correlated with lower salvage rates. Lastly, free flaps to the breast/trunk, fasciocutaneous or osteocutaneous flaps, and those with venous compromise were most associated with higher salvage rates.

Individual factors correlated with salvage outcomes include age and BMI. Age is negatively correlated with FTT salvage outcomes (Figure 2a) but is not significant. This result can be expected, as age is a strong factor in atherosclerotic disease (Jaffer et al., 2002; Nozue et al., 2014). This intrinsic factor impacting vessel flow and clotting propensity is not affected by salvage attempts, and therefore was expected to be correlated to salvage rate in a negative fashion; one cannot simply undo this damage. Similarly, BMI is negatively correlated with many surgical outcomes, including FTT complication rate (Ozturk et al., 2014; Ri et al., 2015). While neither of these analyses were statistically significant, this data does trend in the direction of the established relationships with FTT salvage. Overall, linear regression analysis suggests patients with increased BMI and of greater age are associated with lower salvage rates, and thus nonideal candidates for salvage attempt.

History of previous takeback attempts is significantly associated with salvage failure (Table 3). There are likely many surgical factors involved in this, including accumulative vessel fragility and increased time from index operation. While a history of radiation therapy was not significantly correlated with failed salvage, it did approach significance (*p* = .06), and was significantly associated with failed salvage in the head and neck population (*p* = .028). The impact of radiation on free flap reconstruction is well‐documented (Onoda et al., 2014; Sweeny et al., 2020), and the association with failed salvage likely stems from recipient vessel friability and inflammation creating an overall pro‐thrombotic microenvironment. In general, patients with assumed vessel friability and fragility make nonideal candidates for salvage attempt.

Of the patient‐specific factors, hypercoagulability was the most significant, with patients lacking a history of hypercoagulability being associated with higher salvage rates (Table 3). Hypercoagulability has long been studied regarding FTT outcomes. While the impact is still debated, most studies report higher flap thrombosis rates among those with hypercoagulability (Biben & Atmodiwirjo, 2019; Wang et al., 2012). Therefore, it is natural to expect lower salvage rates in this population when handling microvasculature. Additionally, two studies included in this review (Carney et al., Mirzabeigi et al.) investigated the impact platelet count (PLT) has on FTT salvage outcomes (Carney et al., 2018; Mirzabeigi et al., 2012). The average PLT for free flap salvage failures and successes was 293 and 227, respectively (data not shown). Overall, this suggests that any predilection toward clotting increases one's risk for thrombosis, and therefore an increased risk of recurrent clotting with reexploration of FTT.

One significant finding was that breast free flaps were associated with higher salvage rates (*p* < .00001) (Table 5). Additionally, H&N flaps are closely associated with higher salvage rates compared to extremity flaps, but this was not significant. These relationships are likely due to the primary indication for the FTT and the subsequent physiologic anomalies. Trunk/breast flaps, almost exclusively breast flaps (i.e., DIEP), are either delayed or immediate reconstruction in cancer patients who are deemed medically cleared for this surgery; these procedures are planned (without concurrent systemic trauma) and patients are typically otherwise healthy (Chang, Chang, et al., 2016; Chang, Zhang, et al., 2016; Enajat et al., 2010; Mak & Kwong, 2020; Piwnica‐Worms et al., 2020). In comparison, H&N flaps are predominantly in older male patients with a history positive for tobacco use (Chiu et al., 2017). While these procedures are typically planned as well, either concurrent or prior radiation therapy is commonly used in this population (Onoda et al., 2014; Sweeny et al., 2020). As demonstrated in this review, radiation history is closely associated with FTT salvage failure. Lastly, extremity free flap salvage attempts yielded the lowest success rates (Table 2) when compared to both trunk/breast and H&N (Table 5). Similarly, this is also likely due to the primary population which undergoes extremity reconstruction – a younger male patient population following serious trauma (Cho et al., 2018; Piwnica‐Worms et al., 2020). These patients typically present with polytrauma and multiple concurrent injuries, and are thus likely in a hypovolemic, systemic inflammatory response state with large vessel injury, inducing hypercoagulability and higher PLT counts (Carney et al., 2018; Kloeters et al., 2017; Xiong et al., 2016). As we have shown, patients with a history of hypercoagulability are associated with decreased salvage rates. Additionally, these procedures are not as commonly planned, adding secondary factors impacting salvage, such as time of day, surgeon difference, or surgeon experience, which all likely have some, albeit smaller, effects on salvage outcomes (Chang, Chang, et al., 2016; Chang, Zhang, et al., 2016; Lee & Mun, 2013; Mirzabeigi et al., 2012; Zhao et al., 2020). Overall, these findings confirm the results of Shen et al., which demonstrated that complete failure rates vary by anatomic location, with salvage of breast flaps being the most successful (Shen et al., 2021).

FTT tissue type is associated with salvage procedure outcomes (Table 5). Muscle‐containing flaps (i.e., myocutaneous) are associated with lower FTT salvage rates compared to both osteocutaneous and fasciocutaneous. This was observed statistically when combining osteocutaneous and fasciocutaneous flaps together for analysis. Explanations for this finding are likely based on the metabolic requirements of muscle being higher than skin, fat, and bone (Rojdmark et al., 2002). With a higher metabolic requirement, there is a higher risk of injury with equivalent ischemia time. Additionally, muscle‐based flaps are more commonly used in H&N or extremity reconstruction (Dat et al., 2017); as we have seen, these anatomic locations are associated with lower salvage rates for previously discussed reasons.

Lastly, venous as opposed to arterial compromise and combined venous/arterial compromise is associated with higher FTT salvage rates (Table 5). One explanation for this finding is the sequence in which arterial and venous injury occur. In general, there are two etiologies of delayed (i.e., not during primary anastomosis) arterial thrombosis. One is intrinsic mechanisms related to clotting cascades or recipient vessel damage resulting in “white” (platelet) clot. The second is the more insidious mechanism related to arterial failure after venous outflow obstruction (Eriksson et al., 2005; Halle et al., 2010; Schwarzmaier et al., 2010). Given that arterial failure is often related to patient‐specific factors, (i.e., hypercoagulability, recipient vessel damage) it is assumed that these are not modifiable and thus not impacted by salvage attempt. Furthermore, venous outflow obstruction is often caused by modifiable factors – kinking, relative length mismatch, pedicle redundancy – and as such, they can frequently be corrected at the time of salvage. Interestingly, supercharging does not appear to be one of these modifiable factors associated with salvage success, thus requiring further study to define targets related to venous anastomosis.

Of all potential factors impacting salvage outcomes, time is perhaps the most well‐known and important. The only systematic review studying FTT salvage confirms this general knowledge, with declining salvage rates as time from index operation to compromise increases (Shen et al., 2021). This is well supported throughout the literature and further supported by our systematic analysis (Bui et al., 2007; Chang, Zhang, et al., 2016; Chen et al., 2007; Hyodo et al., 2007; Mirzabeigi et al., 2012; Smit et al., 2007; Winterton et al., 2010; Yii et al., 2001). Because of the way most studies reported data, we analyzed time through correlation plots, relating a study's average “time interval” with the overall study salvage rate (Figure 3). Those studies reporting in “time blocks” were not included in this analysis. Our findings mirror Shen et al: time from index operation to salvage attempt is strongly correlated with study salvage rate in a negative fashion. Explanations as to why time is an important factor impacting salvage outcomes is likely centered on ischemia time. It is well known that a higher ischemia time, such as in organ transplantation, is associated with increased risk of graft failure (Ballestin et al., 2018; Siemionow & Arslan, 2004). Additionally, flap monitoring is likely interlaced. Most centers have flap monitoring as standard of care, with ultrasound being a mainstay of this model. In the present review, 25 of 36 studies reported a monitoring protocol of some capacity, with the majority (18/36) including ultrasound. While multiple methods of monitoring have been studied, including implantable doppler probes and microdialysis, overall results suggest undifferentiating outcomes (Dakpe et al., 2020; Paydar et al., 2010; Rogers et al., 2013; Smit et al., 2010). Ultimately, high frequency clinical monitoring—despite its high cost (Jablonka et al., 2019)—improves accuracy of detecting flap compromise and likely shortens time from detection to salvage (Chae et al., 2015; Frost et al., 2015; Rozen et al., 2010; Soteropulos et al., 2019).

In summary, the failing FTT that is associated with the highest likelihood of salvage is one that is of the trunk/breast without muscle, with venous compromise, without hypercoagulability, without previous takeback attempts, and with a minimal time interval from index operation. Without odds ratios presented in most studies included, we can only offer correlations and associations between factors and salvage success; this is not a study of causality. In the future, studies investigating FTT salvage should summarize data through odds ratios to advance analytic potential. Another limitation of this data was a lack of stratification by salvage method, meaning whether thrombolysis, thrombectomy, or both were utilized. We chose not to include this in our analysis, as choice of method is predicated by surgeon‐perceived severity and other confounding factors—with highly varied responses—and was thus left out (Panchapakesan et al., 2003; Selber et al., 2012). Additionally, we recognize publication bias as potentially skewing these results. However, this is likely not overwhelming, as most factors reported were secondary results in studies, and thus reflect a diverse array of outcomes. Lastly, because we only included articles reporting factors impacting salvage outcomes, we naturally are missing a large sample of overall salvage rate.

Lastly, and in summary, this project was undertaken to provide a data set to serve as a guide in making difficult decisions in challenging circumstances and to add to the microsurgeon's arsenal of educational resources when setting expectations with patients. This is best recapitulated by the words of Professor Wayne Morrison when referring to the “theater of the absurd” that “Not all flaps will work, and futile added hours spent primarily to salvage the surgeon's ego must be resisted.” (Wei & Mardini, 2009) Our hope is that this data will save futile hours and will positively influence both patients and microsurgeons alike.

## 5 CONCLUSIONS

Free flap reconstruction is a versatile surgical modality for plastic and reconstructive surgeons. However, the most feared complication of flap compromise requires costly clinical monitoring and surgical revision through salvage procedures. The present data provides the first detailed summary of both patient‐ and case‐specific factors associated with salvage outcomes. Based on the 36 articles included, the presence of hypercoagulability, increased time from index operation to salvage, anatomic location of flap on the extremities, type of flap including muscle, and the presence of previous takebacks are the strongest factors associated with low salvage rates. Additionally, previous radiation has a significant impact on head and neck flaps salvage success. Salvage operations remain highly variable and create a feared and often misunderstood clinical picture for surgeons and patients alike, and require further data‐driven evaluations to determine guidelines.

## AUTHOR CONTRIBUTIONS

Scott K. Odorico was involved in project inception, project development, article review, data acquisition, manuscript writing, and editing. Katie Reuter Muñoz was involved in project development, article review, data acquisition, and manuscript editing. Peter J. Nicksic was involved in project development, data acquisition, manuscript writing, and editing. Kirsten A. Gunderson was involved in article review, data acquisition, and project development. Kasey Wood was involved in article review, data acquisition, and project development. Zeeda H. Nkana was involved in article review, data acquisition, and project development. Evalina Bond was involved in project inception and development. Samuel O. Poore was involved in project inception, project development, article review, manuscript editing, and overall support.

## Data Availability

The data that support the findings of this study are available from the corresponding author upon reasonable request.
